# Rotenone-induced inner retinal degeneration via presynaptic activation of voltage-dependent sodium and L-type calcium channels in rats

**DOI:** 10.1038/s41598-020-57638-y

**Published:** 2020-01-22

**Authors:** Masaaki Sasaoka, Takashi Ota, Masaaki Kageyama

**Affiliations:** Global Alliances and External Research, Santen Pharmaceutical Co., Ltd., Ikoma-shi, Nara 630-0101 Japan

**Keywords:** Molecular neuroscience, Mechanisms of disease

## Abstract

Rotenone, a mitochondrial complex I inhibitor, causes retinal degeneration via unknown mechanisms. To elucidate the molecular mechanisms of its action, we further characterized a rat model of rotenone-induced retinal degeneration. Intravitreal injection of rotenone (2 nmol/eye) damaged mainly the inner retinal layers, including cell loss in the ganglion cell and inner nuclear layers, which were very similar to those induced by 10 nmol/eye N-methyl-D-aspartate (NMDA). These morphological changes were accompanied by the reduced b-wave amplitude of electroretinogram, and increased immunostaining of 2,4-dinitrophenyl, an oxidative stress marker. Rotenone also downregulated expression of neurofilament light-chain gene (Nfl) as a retinal ganglion cell (RGC) marker. This effect was prevented by simultaneous injection of rotenone with antioxidants or NMDA receptor antagonists. More importantly, voltage-dependent sodium and L-type calcium channel blockers and intracellular calcium signaling modulators remarkably suppressed rotenone-induced Nfl downregulation, whereas none of these agents modified NMDA-induced Nfl downregulation. These results suggest that rotenone-induced inner retinal degeneration stems from indirect postsynaptic NMDA stimulation that is triggered by oxidative stress-mediated presynaptic intracellular calcium signaling via activation of voltage-dependent sodium and L-type calcium channels.

## Introduction

Rotenone is a naturally occurring and broad-spectrum pesticide that inhibits the activity of NADH dehydrogenase in the mitochondrial respiratory chain complex I^1^. Because of this unique biological activity, rotenone has been used as a versatile tool to study involvement of mitochondrial functions and oxidative stress in neuronal cell death. Rotenone causes dopaminergic neurodegeneration that recapitulates key clinical features of Parkinson’s disease, such as nigro-striatal degeneration, formation of Lewy body-like cytoplasmic inclusions, and impaired locomotor activities^2–4^. Rotenone-induced neurodegeneration is also known as an animal model for Leber’s hereditary optic neuropathy (LHON), a rare retinal disease caused by a genetic defect of complex I function^5^. Zhang *et al*.^6^ demonstrated, for the first time, that single intravitreal injection of rotenone reduced the thickness of the ganglion cell layer (GCL). Additional studies reported that the rotenone-reduced GCL thickness was accompanied by retinal ganglion cell (RGC) apoptosis and optic nerve atrophy^7,8^. In contrast, others demonstrated that rotenone had no effect on RGC survival, whereas it caused photoreceptor cell death^9^. Thus, the available results are still controversial, requiring further characterization of a rotenone model of retinal degeneration.

Despite the clinical relevance and utility of this animal model for testing novel therapies, little attention has been paid to the molecular mechanisms underlying rotenone-induced retinal degeneration. However, clues are provided by earlier studies that have tested various therapeutic modalities in this model. Based on its major pharmacological action, oxidative stress is thought to be the key contributor to rotenone-induced retinal damage. In fact, its retinal degenerative effect is accompanied by increased lipid peroxidation^10^ and superoxide production in the mouse retina^7^. Viral delivery of yeast NADH-quinone oxidoreductase, which is equivalent to the mammalian complex I, protected the retina against degeneration and restored visual functions in rodents receiving local administration of rotenone into the brain^11^ and the eye^12^. Additionally, intravitreal injection of methylene blue^10,13^ and oral administration of idebenone^14^, both of which are antioxidants, reduced rotenone-induced RGC loss in mice. More importantly, another study demonstrated that the N-methyl-D-aspartate (NMDA) receptor antagonist memantine dramatically suppressed rotenone-induced RGC death and oxidative stress^15^. These results suggest that oxidative stress and excitotoxicity play major roles in rotenone-induced retinal degeneration. However, it remains largely unknown how rotenone-induced oxidative stress and excitotoxicity cooperate to cause retinal degeneration *in vivo*.

The present study was designed to further characterize retinal degeneration following intravitreal injection of rotenone into the rat eyes and to gain more insights into its molecular mechanisms. Here, we report that intravitreal injection of rotenone causes mainly inner retinal degeneration, which is mediated by oxidative stress and excitotoxicity. Our comprehensive pharmacological approaches demonstrate that rotenone-induced oxidative stress may stimulate postsynaptic NMDA receptors indirectly via activation of presynaptic intracellular calcium signaling by sodium and calcium entry through voltage-dependent sodium and L-type calcium channels, respectively.

## Results

### Morphological and functional changes associated with retinal degeneration induced by intravitreal injection of rotenone

**Figure 1:**
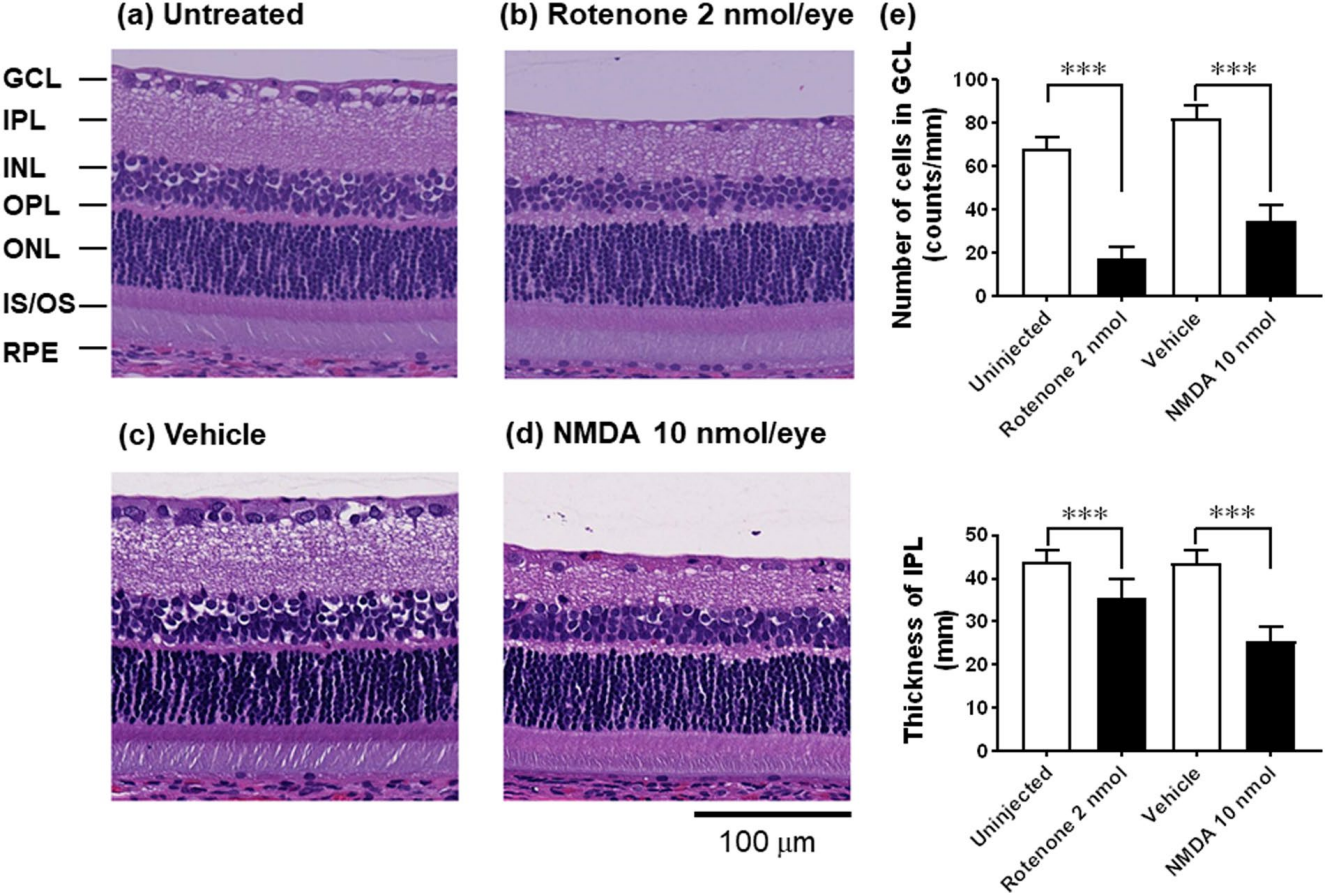
Retinal degeneration induced by rotenone and NMDA in rats. Vehicle (5% DMSO in D-PBS), 2 nmol/eye rotenone or 10 nmol/eye NMDA was intravitreally injected, and each eye was enucleated 3 days following injection. In control animals, the eyes were left uninjected or injected with vehicle. Retinal cross-sections were prepared and stained with hematoxylin and eosin. a-d show the representative images of retinal sections. (**a**) Uninjected (the control for rotenone), (**b**) rotenone, (**c**) vehicle (the control for NMDA), and (**d**) NMDA. GCL: the ganglion cell layer; IPL: the inner plexiform layer; INL: the inner nuclear layer; OPL: the outer plexiform layer; ONL: the outer nuclear layer; IS/OS: the photoreceptor inner and outer segments; RPE: the retinal pigment epithelium. The scale bar shows 100 μm. (**e**) The cell number in GCL (upper panel) and the thickness of IPL (lower panel). Uninjected (open column on the left, n = 8 from 4 animals), rotenone (closed column on the left, n = 7 from 4 animals), vehicle (open column on the right, n = 8 from 4 animals), and NMDA (closed column on the right, n = 8 from 4 animals). Each value represents the mean ± S.D. of 7 to 8 eyes from 4 animals. ***P < 0.001, compared with the respective control group by two-tailed unpaired t-test.

The effect of intravitreal injection of rotenone on the retinal morphology was examined in comparison with that of NMDA, an authentic agent causing inner retinal degeneration via excitotoxicity. Figure 1 shows the representative images of the retinal morphology of the rat eyes 3 days following intravitreal injection of 2 nmol/eye rotenone (Fig. 1b) and 10 nmol/eye NMDA (Fig. 1d). The control eye either remained uninjected (a) or was injected with vehicle (c, Dulbecco’s phosphate buffered solution or D-PBS). Rotenone injection markedly reduced the cell number in GCL and the thickness of the inner plexiform layer (IPL), compared with the uninjected control, and these changes were statistically significant (Fig. 1e, P < 0.0001 for GCL and P = 0.0005 for IPL). The inner nuclear layer (INL), the outer nuclear layer (ONL), and the photoreceptor inner (IS) and outer segments (OS) appeared normal. These features of rotenone-induced inner retinal degeneration were quite similar to those induced by intravitreal injection of NMDA: NMDA injection led to statistically significant reduction in the cell number in GCL and the IPL thickness (Fig. 1d,e, P < 0.0001).

**Figure 2:**
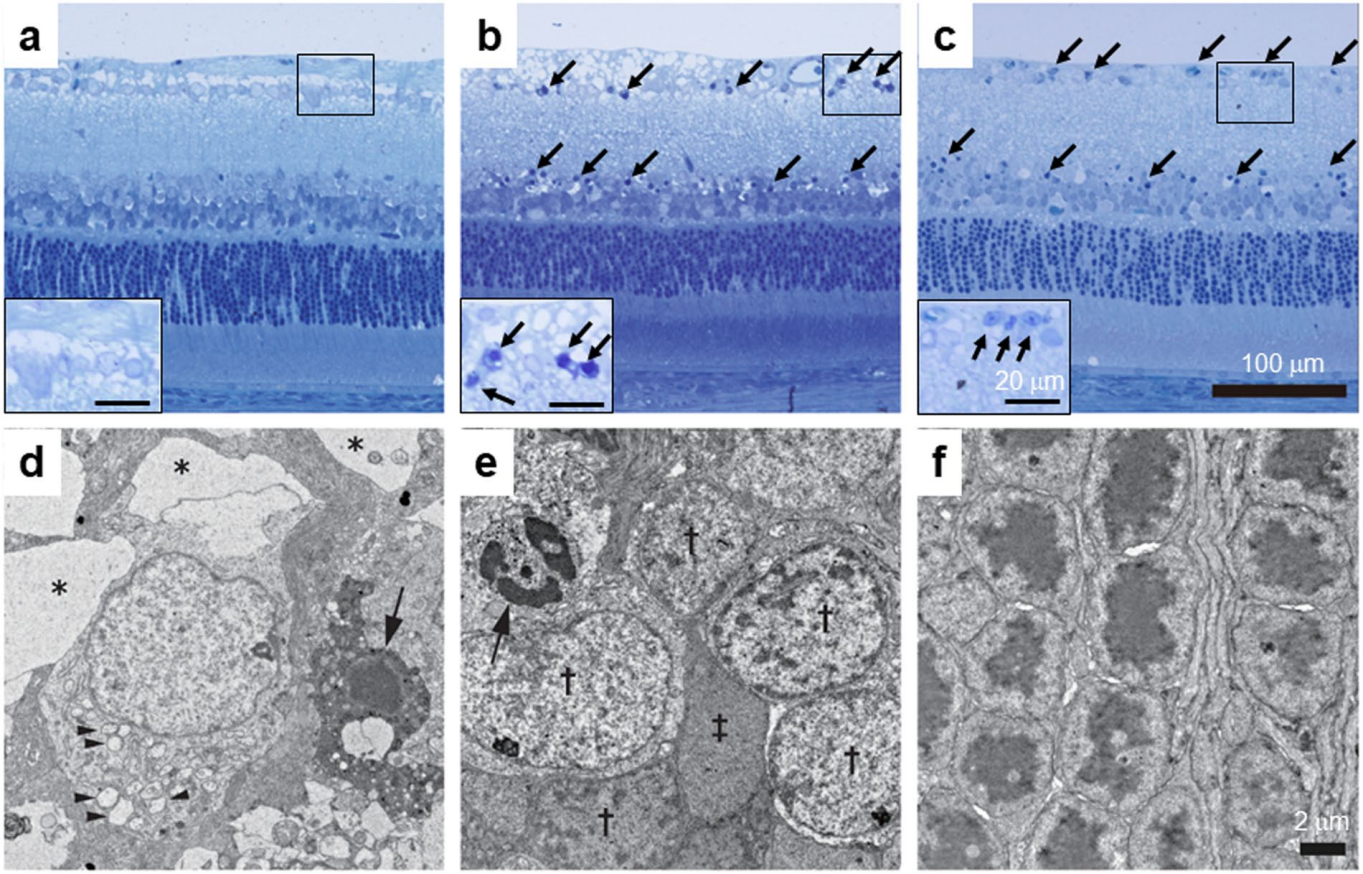
Representative toluidine blue-stained retinal sections and transmission electron microscopic (TEM) images of the retina exposed to rotenone in rats. Either vehicle (50% DMSO in distilled water) or rotenone (2 nmol/eye) was intravitreally injected, and each eye was enucleated 6 or 24 hrs following injection. Retinal cross-sections for toluidine blue staining (**a**–**c**) and ultra-thin sections for TEM (**d**–**f**) were prepared. (**a**) Vehicle (24 hrs after injection), (**b**) rotenone at 6 hrs, and (**c**) rotenone at 24 hrs. The scale bar shows 100 μm. Insets (**a**–**c**): magnified images of the respective rectangular area (the scale bar = 20 μm). Arrows indicate pyknotic cells. (**d**) The ganglion cell layer (rotenone at 6 hrs), (**e**) the inner nuclear layer (rotenone at 6 hrs), and (**f**) the outer nuclear layer (rotenone at 24 hrs). Arrows, asterisk, and arrowheads indicate pyknotic nucleus (**d** and **e**), vacuolation of nerve fiber (**d**), and enlarged granular endoplasmic reticula (**d**), respectively. Dagger and double-dagger symbols indicate normal neuronal cells in the inner nuclear layer and Müller cells, respectively (**e**). Note that photoreceptors appeared normal in (**f**). The scale bar shows 2 μm.

Using toluidine blue staining (Fig. 2a–c) and transmission electron microscopy (TEM, Fig. 2d–f), we further characterized inner retinal degeneration induced by intravitreal injection of 2 nom/eye rotenone. In toluidine blue-stained sections, pyknotic nuclei (solid arrows) were observed predominantly in GCL and INL 6 hrs (Fig. 2b) and 24 hrs following rotenone injection (Fig. 2c). The TEM image (Fig. 2d) revealed that pyknotic nuclei (solid arrow) was accompanied by enlarged granular endoplasmic reticula (arrowheads) and vacuolation of nerve fibers (asterisk) in GCL 6 hrs following rotenone injection. In INL, pyknotic nuclei (solid arrow) was also observed in a subset of neuronal cells (Fig. 2e), but not in other neuronal cells (single daggers) or Müller cells (double dagger). As shown in Fig. 2f, photoreceptor cells remained intact 24 hrs following rotenone injection.

**Figure 3:**
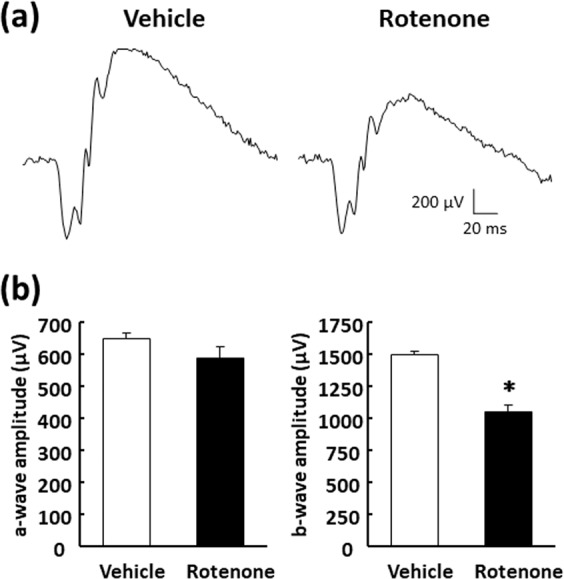
Retinal functional damage induced by rotenone in rats. Vehicle (50% DMSO in distilled water) was intravitreally injected into one eye of each animal, and 2 nmol/eye rotenone was administered into the follow eye. Twenty-four hours following injection, scotopic electroretinograms (ERG) were recorded and the amplitudes of a-wave and b-wave were determined for each ERG recording. (**a**) The representative ERG recordings for the retina exposed to vehicle (left) and rotenone (right). (**b**) Quantitative a-wave (left) and b-wave (right) amplitudes recorded in the vehicle- (open column) and rotenone-injected eyes (closed column). Each value represents the mean ± S.D. of 3 eyes from 3 animals. *P < 0.05, compared with the vehicle control by two-tailed paired t-test.

To determine functional relevance of the rotenone-induced retinal morphological changes, we examined its effect on electroretinogram (ERG) 24 hours following intravitreal injection of 2 nmol/eye rotenone. Figure 3a,b show the representative ERG recordings and the quantitative measurements of the a-wave and b-wave amplitudes, respectively, following vehicle and rotenone injection. In the eyes injected with rotenone, the a-wave amplitude was comparable to that observed in the vehicle injected eyes (left panel in Fig. 3b). The b-wave amplitude was slightly reduced following rotenone injection, and these changes were statistically significantly (right panel in Fig. 3b, P = 0.0324). Thus, the rotenone-induced morphological changes may be accompanied by a defect in signal transmission in the inner retinal layers, but not in the photoreceptor layer.

### Involvement of oxidative stress, proteasome dysfunction and endoplasmic reticulum (ER) stress in rotenone-induced inner retinal degeneration

**Figure 4:**
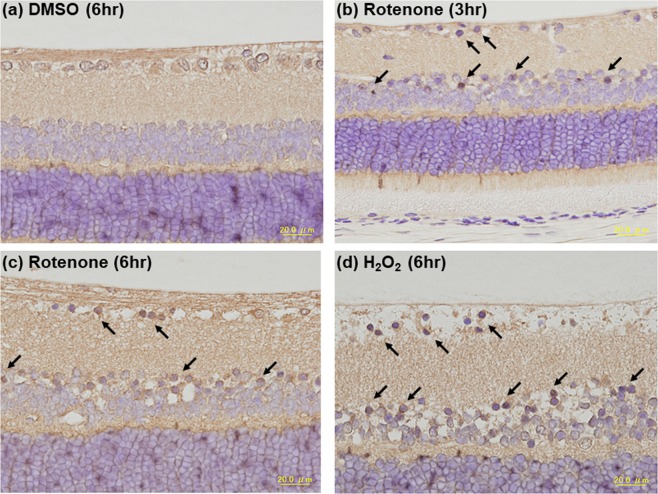
Increased oxidative stress by rotenone and hydrogen peroxide in the rat retina. Vehicle (50% DMSO in distilled water), rotenone (2 nmol/eye) or hydrogen peroxide (1 μmol/eye) was intravitreally injected, and each eye was enucleated 3 or 6 hrs following injection. For each cross-sectioned retina, immunohistochemistry was performed using an antibody against 2,4-dinitropheny (DNP) as a marker for proteins carbonyls (brown). The nuclei were counter-stained with hematoxylin (blue). (**a**) Vehicle (6 hrs after injection), (**b**) rotenone at 3 hrs, (**c**) rotenone at 6 hrs, and (**d**) hydrogen peroxide (H_2_O_2_) at 6 hrs. Arrows show the representative cells with immunopositive signals. The scale bar shows 20 μm.

To elucidate the potential mechanisms underlying rotenone-induced inner retinal degeneration, we performed retinal immunohistochemistry for protein carbonyls, ubiquitin, 20S proteasome and CHOP (also known as GADD153) following intravitreal injection of 2 nmol/eye rotenone. Figure 4 shows the representative image of the retina immunostained with an antibody against 2,4-dinitrophenyl (DNP) as a marker for protein oxidation^16^. DNP-positive staining uniformly distributed throughout the inner retina exposed to vehicle only (Fig. 4a). Three hours following rotenone injection, DNP-positive immunostaining was further increased in cells localized in GCL and INL (Fig. 4b), and maintained at the same level for 6 hrs (Fig. 4c). A similar pattern of increased DNP-positive immunostaining in GCL and INL was observed 6 hrs following intravitreal injection of 1 μmol/eye hydrogen peroxide (Fig. 4d).

**Figure 5:**
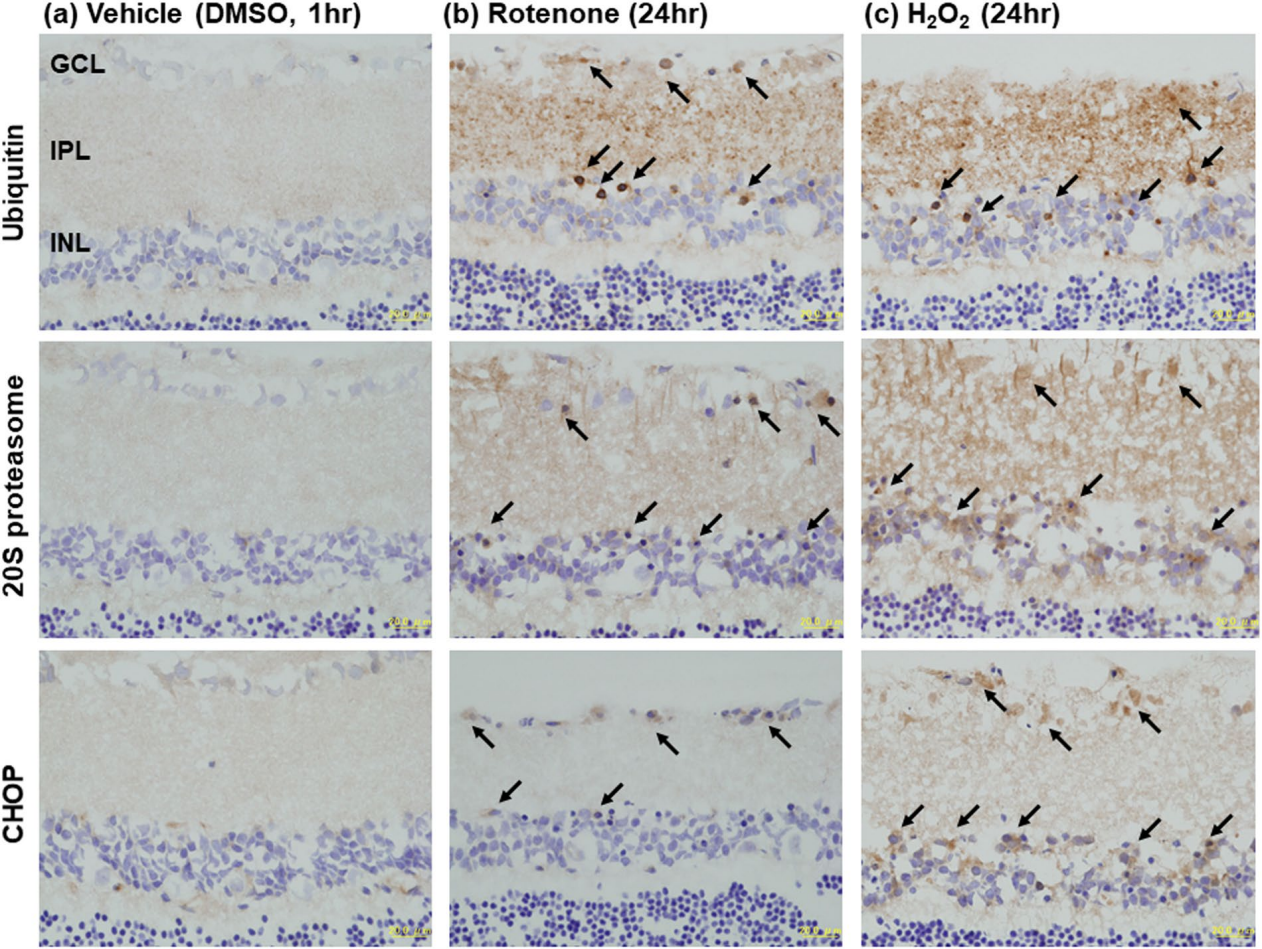
Increased ubiquitin-, 20S proteasome- and CHOP-positive immunohistochemical staining in the rat retina exposed to rotenone and hydrogen peroxide. Vehicle (**a**, 50% DMSO in distilled water), rotenone (**b**, 2 nmol/eye), or hydrogen peroxide (**c**, 1 μmol/eye, H_2_O_2_) was intravitreally injected, and eyes were enucleated 1 and 24 hrs following injection. Each cross-sectioned retina was subjected to immunohistochemical staining for antibodies against ubiquitin (upper panel), 20S proteasome subunit (middle panel) and CHOP (lower panel). Each photograph shows the representative images of cross-sectioned retina following injection. GCL: the ganglion cell layer; IPL: the inner plexiform layer; INL: the inner nuclear layer. Arrows indicate the representative cells with positive immunostaining for each antibody. Each scale bar shows 20 μm. Note that this experiment was performed as part of our previous study published elsewhere (Kageyama *et al*., PLoS One, 2019)^19^. The same control images are used in this figure.

In Fig. 5, the retina was immunostained with antibodies against ubiquitin and 20S proteasome, and CHOP as markers for proteasome function and the ER stress, respectively. In the vehicle-injected eye, immunostaining of any of three marker proteins was faint throughout the retina (Fig. 5a). Twenty-four hours following intravitreal injection, rotenone (2 nmol/eye) remarkably increased ubiquitin- and 20S proteasome-positive immunostaining in GCL, IPL and INL, (Fig. 5b). Similarly, 1 μmol/eye hydrogen peroxide injection led to increased immunostaining of these marker proteins in the same retinal layers at 24 hrs, and the changes were more pronounced (Fig. 5c). Rotenone increased CHOP-positive immunostaining in cells in GCL and INL, and hydrogen peroxide increased it in GCL, IPL and INL. The similar patterns of immunostaining of these 3 proteins were observed at 6 hrs following rotenone injection, although signals were less intense (Supplementary Fig. S1). These results suggest that oxidative stress leads to proteasome dysregulation and increased ER stress in the retina exposed to rotenone.

### Oxidative stress and excitotoxicity as key components of the molecular mechanisms for inner retinal degeneration induced by rotenone

**Figure 6:**
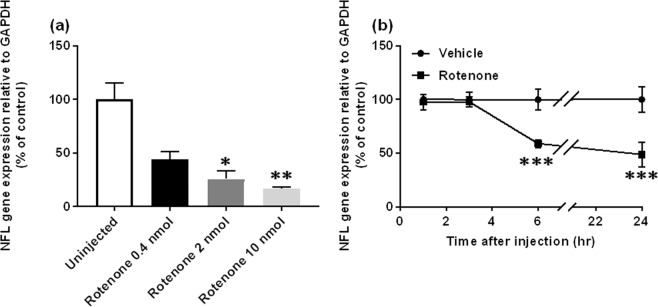
Dose- and time-dependent downregulation of neurofilament light chain gene (Nfl) in the rat retina as a marker for retinal ganglion cells following rotenone injection. In (**a**), rotenone (n = 4 from 2 animals) was intravitreally injected at doses of 0.4 (closed column), 2 (dark grey) and 10 nmol/eye (light grey), and each eye was enucleated 24 hrs following injection. In the control group (open column, n = 4 from 3 animals), the eyes were left uninjected. In (**b**), either vehicle (closed circle, 50% DMSO in distilled water, n = 4 from 2 animals) or 2 nmol/eye rotenone (closed square, n = 4 from 2 animals) was administered into the eyes. One up to twenty-four hours following injection, the retina was isolated and Nfl expression was determined by real-time PCR. The Nfl expression level was normalized to that of Gapdh in an individual retinal sample and is shown as the percentage of the respective control (uninjected group in a and time-matched vehicle control in **b**). Each value represents the mean ± S.D. of 4 eyes from 2–3 animals. *P < 0.05; **P < 0.01; ***P < 0.001, compared with the uninjected and vehicle control groups by Dunn’s (**a**) and Sidak’s multiple comparison tests (**b**), respectively.

For further mechanistic analysis of rotenone-induced inner retinal degeneration, we used the expression of neurofilament light chain gene (Nfl) as a marker for RGC number, as seen in earlier studies^17–19^. This gene was chosen because the reduced cell number in GCL is the most prominent morphological change following rotenone injection. As shown in Fig. 6(a), 24 hours following intravitreal injection at 0.4, 2 and 10 nmol/eye, rotenone reduced Nfl expression in a dose-dependent manner, compared with the uninjected control group (P = 0.0472 and 0.0012 for 2 and 10 nmol rotenone, respectively). At 2 nmol/eye, rotenone did not alter Nfl expression at either 1 or 3 hrs, but reduced it at 6 and 24 hrs post-injection (Fig. 6b). The changes at both time points were statistically significant (P < 0.0001). We also measured the expression of other cell type-specific marker genes (see Supplementary Fig. S2). Rotenone injection dose-dependently reduced the expression of parvalbumin gene (Pvalb), but not tyrosine hydroxylase (Th), which are markers for amacrine cells^20,21^. For bipolar cells, rotenone slightly increased metabotropic glutamate receptor 6 gene (Grm6) expression^21^, whereas it did not alter protein kinase C alpha gene (Prkca) expression^21,22^. The photoreceptor marker opsin 1 gene, short-wave-sensitive (Opn1sw) expression^22^, but not rhodopsin (Rho)^20,22^, was also reduced. Taken together, Nfl expression represents rotenone-induced inner retinal degeneration, and can be used as a surrogate marker for further pharmacological analysis.

**Figure 7:**
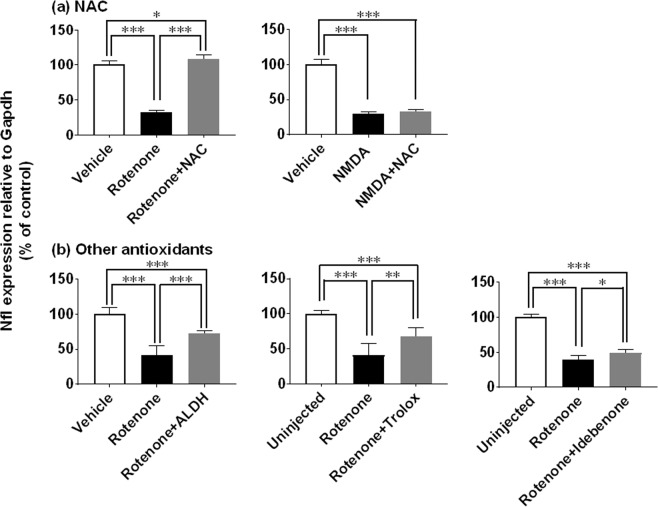
Retinal protective effects of antioxidants against inner retinal degeneration induced by rotenone, but not NMDA, as measured by neurofilament light chain gene (Nfl) expression. Each antioxidant was premixed with either 2 nmol/eye rotenone or 10 nmol/eye NMDA, and simultaneously injected into the vitreous of the rat eyes. In each control group, the eyes were injected with vehicle or left uninjected. Twenty-four hours following injection, the retina was isolated and Nfl expression was determined by real-time PCR. The Nfl expression level was normalized to that of Gapdh in an individual retinal sample and is shown as the percentage of the respective control. (**a**) N-acetyl cysteine (NAC, 500 nmol/eye): open column, vehicle (37.5 or 50% DMSO in distilled water, n = 6 from 3 animals); closed column, rotenone (n = 6 from 3 animal) and NMDA alone (n = 6 from 3 animals); dark grey column, rotenone (n = 6 from 3 animals) or NMDA plus NAC (n = 6 from 3 animals). (**b**) Other antioxidants: open column, vehicle (50% DMSO in distilled water, n = 6 from 3 animals for ALDH) and uninjected (n = 6 from 3 animals for trolox; n = 4 from 2 animals for idebenone); closed column, rotenone alone (n = 5 from 3 animals for ALDH, n = 6 from 3 animals for trolox; n = 4 from 2 animals for idebenone); dark grey column, rotenone plus aldehyde dehydrogenase (ALDH, 0.025U/eye, n = 6 from 3 animals), trolox (50 nmol/eye, n = 6 from 3 animals) or idebenone (25 nmol/eye, n = 4 from 2 animals). Each value represents the mean ± S.D. of 4 to 6 eyes from 2 to 3 animals. *P < 0.05; **P < 0.01; ***P < 0.001, by Tukey’s multiple comparison test.

**Figure 8:**
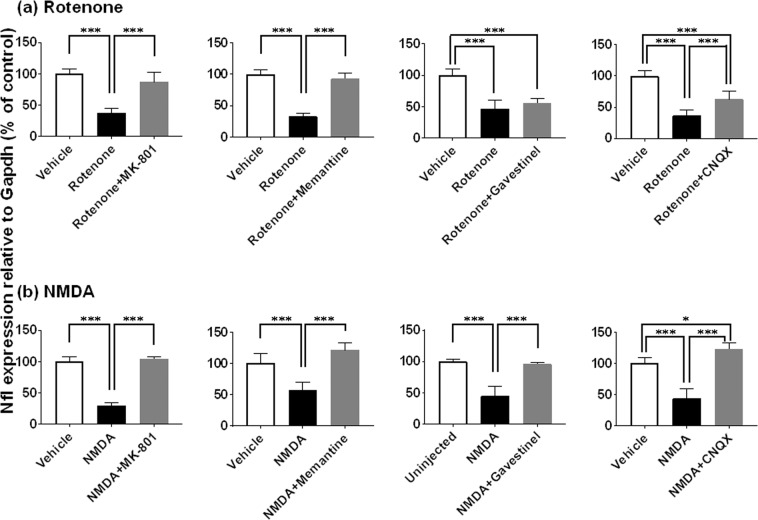
Retinal protective effects of glutamate receptor antagonists against rotenone- and NMDA-induced inner retinal degeneration as measured by neurofilament light chain gene (Nfl) expression. Each antagonist was premixed with either 2 nmol/eye rotenone (**a**) or 10 nmol/eye NMDA (**b**), and simultaneously injected into the vitreous of the rat eyes. In each control group, the eyes were injected with vehicle or left uninjected. Twenty-four hours following injection, the retina was isolated and Nfl expression was determined by real-time PCR. The Nfl expression level was normalized to that of Gapdh in an individual retinal sample and is shown as the percentage of the respective control. (**a**) Rotenone: open column, vehicle (50% DMSO in distilled water, n = 6 from 3 animals); closed column, rotenone alone (n = 8 from 4 animals for MK-801, gavestinel and CNQX; n = 6 from 3 animals for memantine); dark grey column, rotenone plus MK-801 (10 nmol/eye, n = 5 from 3 animals), memantine (100 nmol/eye, n = 6 from 3 animals), gavestinel (15 nmol/eye, n = 6 from 3 animals) or CNQX (50 nmol/eye, n = 6 from 3 animals). (**b**) NMDA: open column, vehicle (distilled water or D-PBS, n = 6 from 3 animals for MK-801 and memantine; n = 4 from 2 animals for CNQX) and uninjected (n = 6 from 3 animals for gavestinel); closed column, NMDA alone (n = 6 from 3 animals for MK-801; n = 5 from 3 animals for memantine; n = 4 from 2 animals for gavestinel and CNQX); dark grey column, NMDA plus MK-801 (10 nmol/eye, n = 5 from 3 animals), memantine (100 nmol/eye, n = 6 from 3 animals), gavestinel (15 nmol/eye, n = 6 from 3 animals) or CNQX (100 nmol/eye, n = 4 from 2 animals). Each value represents the mean ± S.D. of 4 to 8 eyes from 2 to 4 animals. *P < 0.05; ***P < 0.001, by Tukey’s multiple comparison test.

To gain more insight into the molecular mechanisms of its action, we first examined the effects of a variety of antioxidants (Fig. 7) and glutamate receptor antagonists (Fig. 8) on rotenone-induced inner retinal degeneration. As shown in Fig. 7a,b, intravitreal injection of rotenone (2 nmol/eye) and NMDA (10 nmol/eye) resulted in statistically significant decrease in Nfl expression, compared with the respective vehicle and uninjected control group (P < 0.0001). Co-injection of rotenone with N-acetyl cysteine (NAC, 500 nmol/eye) remarkably suppressed rotenone-induced Nfl downregulation (Fig. 7a, P < 0.0001 vs rotenone alone). However, NMDA-induced Nfl downregulation was resistant to NAC co-injection. Simultaneous injection of rotenone with any of 3 different types of antioxidants, aldehyde dehydrogenase (ALDH, 0.025U/eye), trolox (50 nmol/eye) and idebenone (25 nmol/eye), also ameliorated rotenone-induced Nfl downregulation (Fig. 7b, P = 0.0002, 0.0049 and 0.0487, respectively, vs rotenone alone). As expected, we confirmed that co-injection of either typical NMDA receptor antagonist MK-801 (10 nmol/eye) or memantine (100 nmol/eye) with rotenone (Fig. 8a) and NMDA (Fig. 8b) resulted in significant suppression of Nfl downregulation (P < 0.0001, vs rotenone or NMDA alone). The glycine-binding site antagonist gavestinel (15 nmol/eye)^23^ slightly reduced rotenone-induced Nfl downregulation, whereas it significantly suppressed NMDA-induced Nfl downregulation (P < 0.0001, vs NMDA alone). Unexpectedly, the AMPA/kainate receptor antagonist 6-Cyano-7-nitroquinoxaline-2,3-dione (CNQX, 50 and 100 nmol/eye, respectively) substantially reduced Nfl downregulation induced by both rotenone and NMDA (P = 0.0005 and P < 0.0001, vs rotenone and NMDA alone, respectively). These results extend the earlier findings that oxidative stress and excitotoxicity may play primary roles in rotenone-induced inner retinal degeneration.

### Involvement of ion imbalance in inner retinal degeneration induced by rotenone

**Figure 9:**
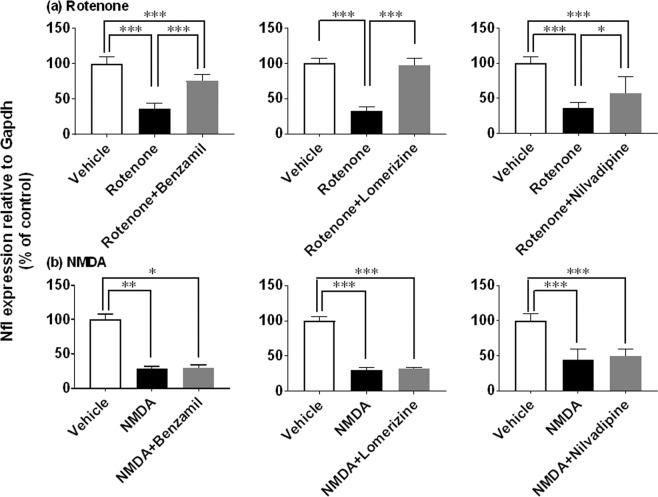
Retinal protective effects of ion channel blockers against inner retinal degeneration induced by rotenone, but not NMDA, as measured by neurofilament light chain gene (Nfl) expression. Each blocker was premixed with either 2 nmol/eye rotenone (**a**) or 10 nmol/eye NMDA (**b**), and simultaneously injected into the vitreous of the rat eyes. Twenty-four hours following injection, the retina was isolated and Nfl expression was determined by real-time PCR. The Nfl expression level was normalized to that of Gapdh in an individual retinal sample and is shown as the percentage of the respective control. (**a**) Rotenone: open column, vehicle (50% DMSO in distilled water, n = 6 from 3 animals); closed column, rotenone alone (n = 8 from 4 animals for benzamil and nilvadipine; n = 6 from 3 animals for lomerizine); dark grey column: rotenone plus benzamil (25 nmol/eye, n = 6 from 3 animals), lomerizine (50 nmol/eye, n = 6 from 3 animals), or nilvadipine (5 nmol/eye, n = 6 from 3 animals). (**b**) NMDA: open column, vehicle (50% DMSO in distilled water or distilled water, n = 6 from 3 animals for benzamil and lomerizine; n = 4 from 2 animals for nilvadipine); closed column, NMDA alone (n = 6 from 3 animals for benzamil and lomerizine; n = 4 from 2 animals for nilvadipine); dark grey column, NMDA plus benzamil (25 nmol/eye, n = 6 from 3 animals), lomerizine (50 nmol/eye, n = 6 from 3 animals) or nilvadipine (0.05 nmol/eye, n = 4 from 2 animals). Each value represents the mean ± S.D. of 4 to 8 eyes from 2 to 4 animals. *P < 0.05; **P < 0.01; ***P < 0.001, by Tukey’s or Dunn’s multiple comparison test.

**Figure 10:**
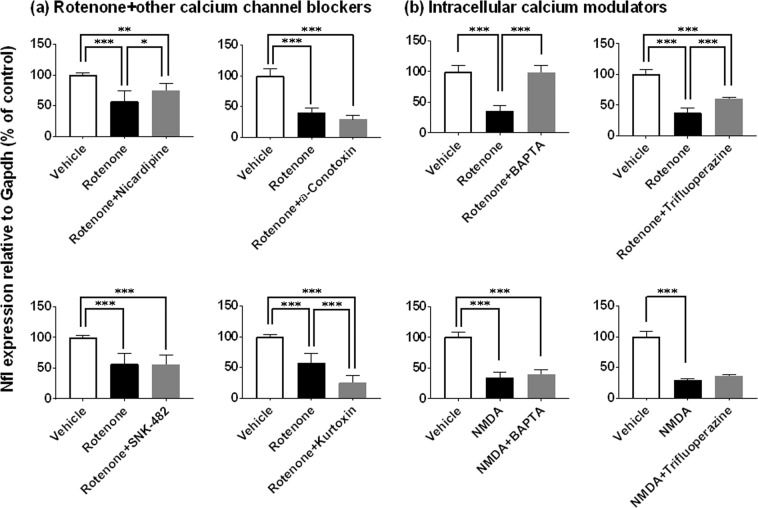
Retinal protective effects of calcium modulators against inner retinal degeneration induced by rotenone, but not NMDA, as measured by neurofilament light chain gene (Nfl) expression. Each modulator was premixed with either 2 nmol/eye rotenone (a and upper panel in b) or 10 nmol/eye NMDA (lower panel in b), and simultaneously injected into the vitreous of the rat eyes. Twenty-four hours following injection, the retina was isolated and Nfl expression was determined by real-time PCR. The Nfl expression level was normalized to that of Gapdh in an individual retinal sample and is shown as the percentage of the respective control. (**a**) Rotenone plus other calcium channel blockers: open column, vehicle (50% DMSO in distilled water, n = 6 from 3 animals for nicardipine, SNX-482 and kurtoxin; n = 8 from 4 animals for ω-conotoxin); closed column, rotenone alone (n = 6 from 3 animals for nicardipine, SNX-482 and kurtoxin; n = 7 from 4 animals for ω-conotoxin); dark grey column, rotenone plus nicardipine (10 nmol/eye, n = 7 from 4 animals), ω-conotoxin (5 nmol/eye, n = 6 from 3 animals), SNX-482 (0.625 nmol/eye, n = 6 from 3 animals) or kurtoxin (0.4 nmol/eye, n = 6 from 3 animals). (**b**) Rotenone (upper panel) or NMDA (lower panel) plus intracellular calcium signaling modulators: open column, vehicle (10 or 50% DMSO in distilled water, n = 6 from 3 animals); closed column, rotenone (n = 8 from 4 animals) and NMDA alone (n = 5 from 3 animals for BAPTA; n = 6 from 3 animals for trifluoperazine); dark grey column, rotenone and NMDA plus BAPTA (125 nmol/eye, n = 6 from 3 animals) or trifluoperazine (25 nmol/eye, n = 6 from 3 animals). Each value represents the mean ± S.D. of 5 to 8 eyes from 3 to 4 animals. *P < 0.05; **P < 0.01; ***P < 0.001, by Tukey’s or Dunn’s multiple comparison test.

Next, we asked whether altered ion channel activities and intracellular calcium signaling might mediate rotenone- and NMDA-induced inner retinal degeneration. To this end, we used a variety of pharmacological inhibitors and modulators of this signaling pathway (Figs. 9 and 10). As shown in Fig. 9a, co-injection of rotenone (2 nmol/eye) with the sodium channel blocker benzamil (25 nmol/eye) led to statistically significant suppression of rotenone-induced Nfl downregulation (P < 0.0001, vs rotenone alone). Similarly, both of L-type calcium channel blocker lomerizine (50 nmol/eye, P < 0.0001) and nilvadipine (5 nmol/eye, P = 0.0408) significantly prevented rotenone-induced Nfl downregulation. In contrast, any of these channel blockers did not modify NMDA (10 nmol/eye)-induced Nfl downregulation (Fig. 9b). These results clearly indicate that activation of voltage-dependent sodium and L-type calcium channels may be required for inner retinal degeneration induced by rotenone, but not NMDA.

We further examined the effects of other types of voltage-dependent calcium channel blockers and intracellular signaling modulators on rotenone-induced inner retinal degeneration. Like lomerizine and nilvadipine, the L-type calcium channel blocker nicardipine (10 nmol/eye, P = 0.0337 vs rotenone alone) significantly suppressed rotenone-induced Nfl downregulation (Fig. 10a). Any of the N-type calcium channel blocker ω-conotoxin (5 nmol/eye), the R-type blocker SNX-482 (0.625 nmol/eye) or the T-type blocker kurtoxin (0.4 nmol/eye) did not prevent rotenone-induced Nfl downregulation. As shown in Fig. 10b, both of the calcium chelator BAPTA (125 nmol/eye) and the calmodulin inhibitor trifluoperazine (25 nmol/eye) significantly ameliorated rotenone-induced Nfl downregulation (P < 0.0001, vs rotenone alone). However, both agents failed to modify NMDA-induced Nfl downregulation. Taken together, ion entry through voltage-dependent sodium and L-type calcium channels, and subsequent intracellular calcium signaling at presynaptic terminals may initiate rotenone-induced inner retinal degeneration.

**Figure 11:**
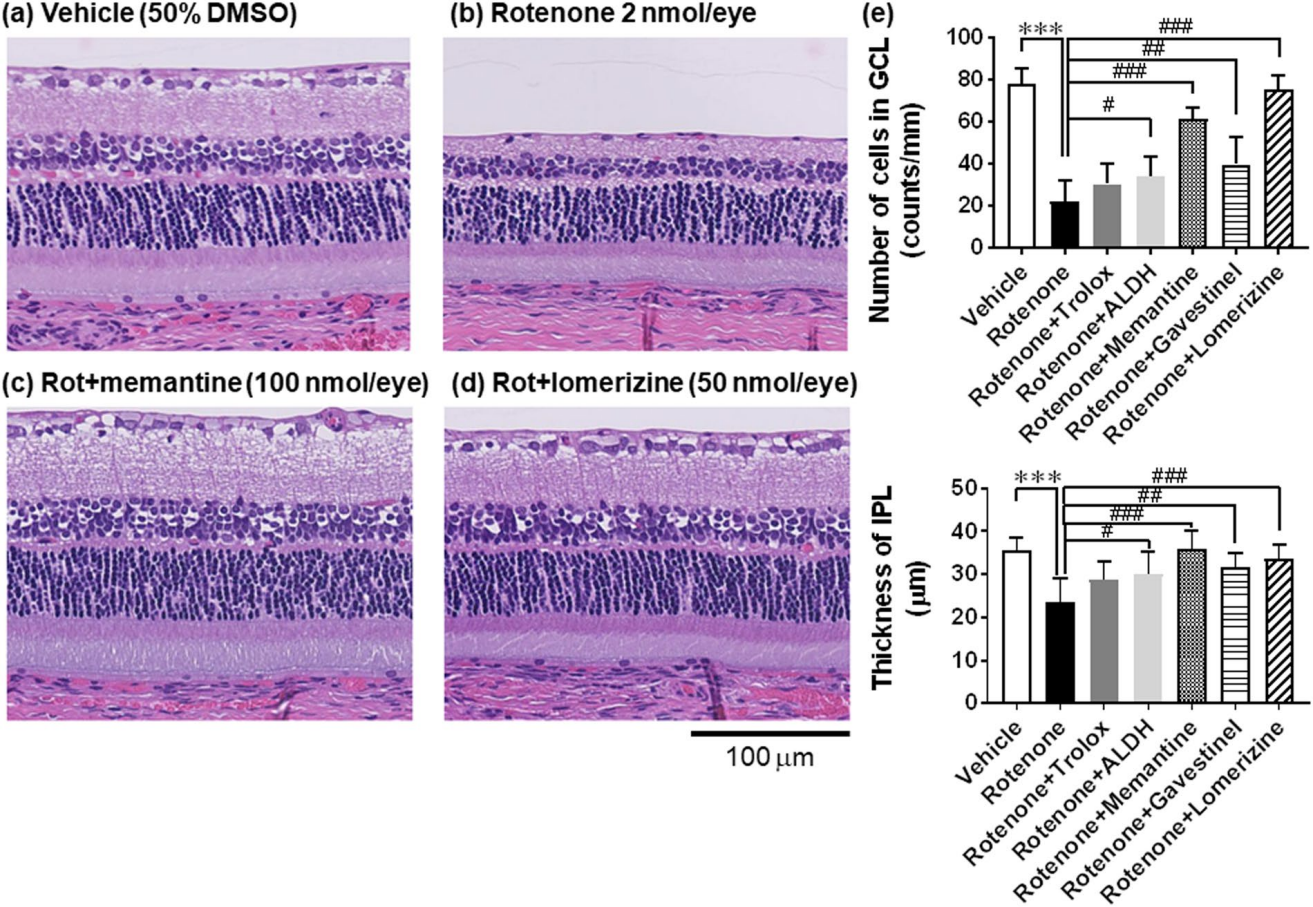
Morphological retinal protection by various pharmacological agents against inner retinal degeneration induced by rotenone. Each agent was premixed with 2 nmol/eye rotenone, and simultaneously injected into the vitreous of the rat eyes. Seven days following injection, each eye was enucleated, retinal cross-sections were prepared, and stained with hematoxylin and eosin. a-d show the representative images of retinal sections. (**a**) Vehicle (50% DMSO), (**b**) rotenone, (**c**) rotenone plus 100 nmol/eye memantine, and (**d**) rotenone plus 50 nmol/eye lomerizine. The scale bar shows 100 μm. See Fig. 1 for the detailed information about the retinal layers. (**e**) The cell number in the ganglion cell layer (GCL, upper panel) and the thickness of the inner plexiform layer (IPL, lower panel). Vehicle (open column, n = 9 from 5 animals), rotenone alone (closed, 10 from 5 animals), rotenone plus trolox (dark grey, 50 nmol/eye, n = 7 from 4 animals), aldehyde dehydrogenase (ALDH, light grey, 0.025 U/eye, n = 7 from 4 animals), memantine (stippled, 100 nmol/eye, n = 6 from 4 animals), gavestinel (horizontal stripped, 15 nmol/eye, n = 5 from 3 animals) and lomerizine (hatched, 50 nmol/eye, n = 7 from 4 animals). Each value represents the mean ± S.D. of 5 to 10 eyes from 3–5 animals. ***P < 0.001, by two-tailed unpaired t-test, compared with vehicle. ^#^P < 0.05; ^##^P < 0.01; ^###^P < 0.001, by Dennett’s multiple comparison test, compared with rotenone alone.

Finally, our histological analysis (Fig. 11) confirmed the results obtained with Nfl expression. Namely, simultaneous intravitreal injection of 2 nmol/eye rotenone with either memantine (100 nmol/eye, Fig. 11c) or lomerizine (50 nmol/eye, Fig. 11d) remarkably prevented inner retinal damage induced by rotenone alone (Fig. 11b). The retinas of the eyes injected with memantine or lomerizine were morphologically similar to those injected with vehicle (Fig. 11a) and appeared to be normal. Their protective effects reached statistical significance (Fig. 11e, P = 0.0002 for lomerizine IPL; P < 0.0001 for the rest, vs rotenone alone). Gavestinel (15 nmol/eye) showed moderate, but statistically significant, inner retinal protection against rotenone-induced damage (P = 0.0053 and 0.0096 for GCL and IPL, respectively). Similarly, co-injection of rotenone (2 nmol/eye) with ALDH (0.025 u/eye) resulted in statistically significant protection against rotenone-induced inner retinal damage (P = 0.0415 and 0.0211 for cell number in GCL and IPL, respectively). Co-injection with trolox (50 nmol/eye) also suppressed rotenone-induced cell loss and reduced thickness of IPL to the same extent as did ALDH, but this effect did not reach statistical significance. The degree of suppression by each agent of rotenone-induced inner retinal degeneration measured by histological analysis was generally consistent with that by Nfl measurements.

## Discussion

The present study further extends our understanding of features of rotenone-induced retinal degeneration in the following ways: 1) intravitreal injection of rotenone into the rat eyes demonstrates morphological and functional damage mainly in the inner retinal layers, including loss of cells in GCL, 2) rotenone-induced inner retinal degeneration is accompanied by increased immunostaining of marker proteins for oxidative stress, proteasome and ER stress, and 3) intracellular calcium signaling and excitotoxicity following sodium and calcium entry through voltage-dependent channels mediate rotenone-induced inner retinal degeneration. Our study provides a robust and versatile rat model of inner retinal degeneration, which is reproducibly induced by intravitreal injection of rotenone and simply quantified by retinal Nfl expression.

It still remains unclear which retinal cell types are vulnerable to rotenone. Particularly, the outcomes following systemic administration of rotenone are quite variable. For instance, repeated intraperitoneal injection of rotenone led to no noticeable retinal morphological changes in the rat eyes^24^. In contrast, subcutaneous administration of rotenone caused outer retinal degeneration, but not the inner retina^9^, whereas intraperitoneal rotenone caused RGC and photoreceptor cell death^8^. As pointed out in the earlier study^9^, this discrepancy may be due simply to different regimens of systemic rotenone administration. In our study, intravitreal injection of rotenone resulted in reproducible inner retinal degeneration, including morphological, functional and molecular changes limited to GCL, IPL and INL, except for reduced Opn1sw expression. This is consistent with the results of earlier studies in the mouse eyes receiving intravitreal rotenone injection^6,25,26^. It is unlikely that its tissue distribution may be limited to the inner retina following intravitreal injection, because lipophilic compounds like rotenone can easily penetrate into the retina, and diffuse into the choroidal circulation^27^. Given the fact that the results with intravitreal injection of rotenone are reproducible among different laboratories, intravitreal delivery seems to be an optimal route of administration to study the molecular mechanisms of retinal action of rotenone.

Another major finding of this study was that rotenone-induced inner retinal degeneration resembles morphologically and functionally to that by NMDA^28^. However, our pharmacological analysis reveals that the molecular mechanisms of their actions are not identical. The key difference is that four chemically and pharmacologically different antioxidants, NAC^29^, the water soluble vitamin E derivative Trolox^30^, ALDH^31^, and idebenone^32^, suppressed rotenone-induced retinal degeneration, whereas NAC failed to prevent NMDA-induced damage. These retinal protective effects of antioxidants are consistent with earlier pharmacological findings^10,13,14^. We also found that rotenone-induced retinal damage was accompanied by increased protein carbonyl-positive immunostaining in the retina, as seen in other studies using dihydroethidium as an oxidative stress indicator^7,15^. The similarity between rotenone- and NMDA-induced inner retinal degeneration is their susceptibility to NMDA receptor antagonists. Namely, MK-801 and memantine provided full retinal protection against rotenone- and NMDA-induced damage, which is consistent with an earlier observation^15^. The glycine-binding site antagonist gavestinel also prevented rotenone- and NMDA-induced retinal damage. Surprisingly, the AMPA/kainate receptor antagonist CNQX significantly reduced retinal damage induced by both chemicals. This may be because CNQX acts as a glycine-binding site antagonist, like gavestinel^33,34^. Interestingly, their retinal protective effects against rotenone-induced damage were milder than those against NMDA-induced damage, suggesting that NMDA receptor activation equally contributes to rotenone- and NMDA-induced inner retinal degeneration, but the regulatory modulation at the glycine-binding site is somehow different. Collectively, we provide further evidence that oxidative stress and excitotoxicity may play crucial roles in rotenone-induced inner retinal degeneration.

A question arises regarding the interplay between oxidative stress and NMDA receptor activation in rotenone-induced inner retinal degeneration. To address this question, we hypothesized that rotenone-induced oxidative stress might increase presynaptic intracellular calcium concentrations and glutamate and glycine release, leading to postsynaptic stimulation of NMDA receptors (see Supplementary Fig. S3). This putative presynaptic mechanism is quite similar to that for retinal ischemia/reperfusion injury where energy depletion leads to increased glutamate and glycine release and excitotoxicity^35,36^. To support this hypothesis, we provide the following pharmacological evidence: First, antioxidants suppressed inner retinal degeneration induced by rotenone, but not NMDA, suggesting presynaptic roles for oxidative stress in rotenone-induced retinal damage. Second, a voltage-dependent sodium channel blocker (benzamil) and L-type calcium channel blockers (lomerizine, nilvadipine and nicardipine) remarkably reduced rotenone-induced retinal damage, whereas they had no effect on that of NMDA. In the retina, voltage-dependent sodium and L-type calcium channels primarily regulate sodium and calcium entry into synaptic terminals, respectively^37,38^. Oxidative stress activates these voltage-dependent channels directly and/or indirectly via inhibition of sodium-potassium ATPase^39–42^. Furthermore, it is well-documented that rotenone increases intracellular calcium concentrations via activation of L-type voltage-dependent calcium channels in neuronal cells^43–46^. Thus, rotenone-induced retinal oxidative stress may activate presynaptic voltage-dependent sodium and L-type calcium channels. Finally, an extracellular calcium chelater (BAPTA) and a calmodulin inhibitor (trifluoperazine) also protected the retina against rotenone-induced damage, but not NMDA. Because the calcium/calmodulin system orchestrates neurotransmitter release from synaptic terminals^47^, it is likely that rotenone-induced oxidative stress may stimulate neurotransmitter release via activation of presynaptic calcium/calmodulin signaling. Taken together, we conclude that rotenone-induced oxidative stress may cause inner retinal degeneration via increased neurotransmitter release following activation of presynaptic intracellular calcium signaling, and subsequent postsynaptic NMDA receptor stimulation.

It is likely that RGC, bipolar cells or photoreceptors may be the site where glutamate is excessively released in response to rotenone, because they are primary excitatory glutamatergic neurons in the retina and L-type voltage-dependent calcium channels exist in RGC, bipolar cells, and rod and cone photoreceptors, as reviewed by Van Hook *et al*.^48^. Given that morphological and functional damage were confined to the inner retina including INL, but not photoreceptors, and that some cells in INL are oxidative stress marker-immunopositive, bipolar cells may be the major target for rotenone-induced oxidative stress and subsequent voltage-dependent sodium and L-type calcium channel activation. Consistently, only L-type voltage-dependent calcium channel blockers significantly suppressed rotenone-induced inner retinal degeneration. Particularly, lomerizine was most effective in prevention of rotenone-induced retinal damage, whereas N-type (ω-conotoxin), R-type (SNX-482) or T-type blocker (kurtoxin) was virtually ineffective. Although synapses of RGC do not exist within the retina, we cannot exclude the possibility that RGC somata may be another target for rotenone, because they possess L-type calcium channel and were oxidative stress marker-positive. In fact, it was reported that ectopic glutamate release from RGC somata (and/or their axons) may contribute to optic nerve damage in an experimentally induced mouse glaucoma model^49^. In this study, we did not measure vitreous glutamate and glycine concentrations following rotenone injection. However, it is still unclear whether retinal excitotoxicity may always need a detectable increase in vitreous amino acid concentrations, because excitotoxic retinal degeneration is induced without significant changes in vitreous glutamate or glycine concentrations in a primate model of retinal ischemia^50^ and glutamate transporter knockout mice^51^. Instead, we provide indirect evidence supporting increased glutamate and glycine release: glycine-binding site antagonists (gavestinel and CNQX) reduced rotenone-induced retinal damage to a lesser extent than NMDA-induced damage, suggesting that increased glycine release by rotenone may counteract their antagonism of postsynaptic NMDA receptors. Thus, rotenone-induced oxidative stress may increase local glutamate concentrations at the presynaptic terminals of bipolar cells and/or RGC somata, resulting in excitotoxicity confined to the inner retina.

Our finding that rotenone- and hydrogen peroxide-induced oxidative stress was accompanied by increased retinal immunostaining of ubiquitin, 20S proteasome and CHOP implies potential roles for dysregulated proteasome function and/or ER stress in oxidative stress-induced inner retinal degeneration. We also found that either ER stress inhibitor (salubrinal, 12.5 nmol/eye) or unfolded protein response modulators (the XBP-1 inhibitor ansatrienin A, 1 nmol/eye; the GSK 3b inhibitor TDZD-8, 1 and 20 nmol/eye) had no effect on rotenone-induced retinal degeneration (see Supplementary Fig. S4). This result suggests that ER stress does not act as an effector to trigger cell death in rotenone-induced retinal degeneration, even though CHOP was upregulated. In contrast, proteasome may contribute to rotenone-induced inner retinal degeneration, because we previously reported that, like rotenone and hydrogen peroxide, the small molecular chemical proteasome inhibitor MG-262 caused a very similar pattern of inner retinal degeneration, which was accompanied by increased immunostaining of these three proteins^19^. Consistently, rotenone was reported to promote formation of cytoplasmic inclusion bodies enclosing ubiquitin and α-synuclein aggregates associated with dopaminergic neuronal degeneration^2^. Oxidized proteins inhibit proteasome activity^29,52^ and rotenone-reduced proteasome activity is tightly correlated with its cytotoxic effect in cultured neuroblastoma cells^53^. Interestingly, unlike rotenone, MG-262-induced retina damage is virtually resistant to any of tested pharmacological agents, including antioxidants, voltage-dependent channel blockers, NMDA receptor antagonists and ER stress modulators^19^. Therefore, proteasome dysfunction may play roles downstream from postsynaptic NMDA receptor activation in rotenone-induced retinal damage. Further studies are underway to clarify the exact downstream events following NMDA receptor activation and chemical proteasome inhibition, leading to inner retinal degeneration.

In conclusion, the present study provides compelling evidence demonstrating that intravitreal injection of rotenone to the rat eyes causes reproducible inner retinal degeneration via increased oxidative stress and excitotoxicity. Furthermore, we delineate the underlying molecular mechanisms that rotenone-induced oxidative stress activates presynaptic voltage-dependent sodium and L-type calcium channel. Activated intracellular calcium signaling may locally stimulate glutamate release from presynaptic terminals, leading to postsynaptic NMDA receptor stimulation and subsequent inner retinal degeneration. Our study provides a convenient and versatile animal model of rotenone-induced inner retinal degeneration, which may be useful for discovery of novel therapeutic modalities for treatment of various forms of inner retinopathy associated with energy consumption imbalance such as LHON.

## Materials and Methods

### Reagents

Rotenone, NMDA, Trolox, and ALDH, MK-801, CNQX, benzamil, nicardipine, BAPTA (1,2-bis(o-aminophenoxy)ethane-N,N,N′,N′-tetraacetic acid), trifluoperazine, and TDZD-8 (4-Benzyl-2-methyl-1,2,4-thiadiazolidine-3,5-dione) were purchased from Sigma-Aldrich, Corp. (St Louis, MO, USA). NAC, idebenone, and CNQX were obtained from Wako Pure Chemical Industries, Ltd. (Osaka, Japan). The other chemicals were provided by the respective vendors: memantine (Sequoia, Pangbourne, UK), gavestinel (Tocris, Avonmouth, Bristol, UK; Axon Medchem BV, Groningen, Netherlands), lomerizine (Organon, Roseland, NJ, USA), nilvadipine (Nivadil, Astellas Pharma Inc, Tokyo), kurtoxin, SNX-482 and ω-conotoxin (Peptide Institute, Osaka, Japan), salubrinal (Tocris, Avonmouth, Bristol, UK), and ansatrienin A (Enzo Life Sciences, Farmingdale, NY, USA). All chemicals were dissolved in distilled water or DMSO to prepare a stock solution and diluted with D-PBS or distilled water to obtain a given final concentration. Doses for each chemical were chosen, based on their *in vitro* activities and the vitreous volume of the rat eye (60 μl)^27^.

### Animals and intravitreal injections

All animals were treated in compliance with the ARVO statement for the Use of Animals in Ophthalmic and Vision Research. We also complied with “Basic Policies for the Conduct of Animal Experiments in Research Institutions” issued by the Ministry of Health, Labour and Welfare, Japan (2006), and “The Guidelines for Proper Conduct of Animal Experiments” published by the Science Council of Japan (2006). All experimental procedures were approved and monitored by the Institutional Animal Care and Use Committee of Santen Pharmaceutical. Every effort was made to avoid unnecessary use of laboratory animals. Adult male Sprague-Dawley rats (190–240 g) were purchased from Japan SLC, Inc. (Hamamatsu, Japan). The environment was kept at 23 ± 3 °C with a 12-hour light and a 12-hour dark cycle. All rats were allowed food and water ad libitum, and they were acclimatized to the environment for at least 1 week prior to the experiment. Each rat was anesthetized with inhalation of isoflurane (3.5% for induction and 2.5% for maintenance). Intravitreal injections were made via a 33-G needle connected to a 25 μL microsyringe (Hamilton company, Reno, NV, USA). The needle penetrated the eye from the nasal sclera at ~1.5 mm posterior to the limbus, and was inserted toward the optic disc to a depth of ~2.5 mm. Both eyes of each animal received a single injection of 5-μl solution containing vehicle, rotenone or NMDA at a given dose. For concomitant injection of either rotenone or NMDA with any of other chemicals, both chemicals were premixed and a 5-μl aliquot of resultant solution was administered in the same way as described above. All injections were performed under a binocular microscope and care was taken not to injure the lens or retina during the procedure. As seen in earlier studies^54,55^, a bilateral approach was taken to minimize the number of animals sacrificed for this study. At a given time point following intravitreal injection of vehicle, rotenone and other chemicals, the animals were intraperitoneally administered excess dose of pentobarbital and the eyes were isolated. They were subjected to further assays as described in the sessions below.

### Histological evaluation

The isolated eyes were fixed in 2% paraformaldehyde-2.5% glutaraldehyde (Wako Pure Chemical Industries, Ltd., Osaka, Japan). After anterior segments and lenses were removed from the eyes, posterior segments (eye cups) were rinsed with water, dehydrated, and embedded in paraffin. Eight horizontal sections of eye cups through the optic disc were prepared at 3-μm thickness per each retina, and stained with hematoxylin and eosin. The whole image of eight sections for each eye was scanned with a fully automated digital slide scanner (NanoZoomer Digital Pathology™, Hamamatsu Photonics K., Shizuoka, Japan). Out of eight sections, three were used for further histological evaluation. The number of cells in GCL and the thickness of IPL were determined on each image including the 800 μm width of the retina starting at a distance of 700 µm from the center of the optic disk. The values were averaged among three sections as the representative value for each eye.

For toluidine blue staining and TEM, the posterior pole of the eyeball was fixed in 2.5% glutaraldehyde and 1% osmium tetroxide, dehydrated, and embedded in epoxy resin (Quetol 812; Nissin EM Co., Tokyo, Japan). Semi-thin sections were cut, and stained with toluidine blue for light microscopy. Ultra-thin sections were obtained essentially in the same way, and stained with uranyl acetate and lead citrate. These sections were observed under an H 7600 electron microscope (Hitachi High-Technologies Co., Tokyo).

### ERG recording

Following injection of vehicle or rotenone into the eyes of each rat, animals were subjected to dark adaptation for at least 4 hours in an electrically shielded room. The animals were intramuscularly injected with a 7:1 mixture of ketamine at 1 ml/kg (Ketalar intramuscular 500 mg; Sankyo Yell Yakuhin Co., Ltd., Tokyo, Japan) and xylazine (Selactar; Bayer Ltd., Tokyo, Japan). The pupil of each animal was dilated with a topical application of both phenylephrine hydrochloride and tropicamide (Mydrin-P; Santen Pharmaceutical Co., Ltd., Osaka, Japan), and the cornea was anesthetized with topical oxybuprocaine hydrochloride (Benoxil Ophthalmic Solution 0.4%; Santen Pharmaceutical Co., Ltd., Osaka, Japan). A contact lens-recording electrode assembled with a white light-emitting diode (LED) stimulator was placed onto the cornea. Reference electrode and ground electrode wires were inserted into the nose and tail, respectively. The cornea was intermittently irrigated with a balanced salt solution to maintain an adequate contact with the electrode, and to prevent exposure keratopathy. Following flashlights by a photostimulator (LS-W; Mayo, Nagoya, Japan), the scotopic ERG was recorded with low- and high-frequency cut-offs at 0.5 and 1.0 kHz (Neuropack S1; Nihon Kohden, Tokyo, Japan). The amplitudes of the recorded a- and b-waves were measured for each eye and averaged in each injection group.

### Immunohistochemistry

The eyes were fixed in a methacarn solution containing 60% methanol, 30% acetic acid, and 10% chloroform. Eye cups were rinsed with 70% ethanol, and embedded in paraffin. Horizontal sections of eye cups through the optic disc at 3-μm thickness were prepared. According to the manufacturer’s instruction, immunohistochemical staining for DNP was performed using Protein Carbonyls Immunohistochemical Staining Kit (Cat No. SML-ROIK04-EX, Shima Laboratories, Co., Ltd., Tokyo, Japan) to detect oxidized proteins in the retina. Briefly, the sections were incubated sequentially with a DNPH solution for 30 min, an anti-DNP antibody (1:400, rabbit polyclonal) for 1 hour, and a Fab-protein complex labeled with peroxidase (Simple Stain Rat MAX PO; Nichirei Biosciences, Inc., Tokyo, Japan) for 1 hour at room temperature. The sections were then stained with diaminobenzidine and counterstained with hematoxylin tetrahydrochloride. For a negative control, the sections were incubated in a 2% HCl solution without DNPH for 30 min before the primary antibody was added.

For the rest of immunohistochemistry, the experiment was performed as part of the previous study published and described elsewhere in details^19^. Briefly, eyes were fixed in a 4% paraformaldehyde solution, embedded in OTC compound, and frozen. Serial cross sections (4-μm thickness) of the retina through the optic disk were prepared and incubated with a primary antibody against ubiquitin (1:200, rabbit polyclonal, Cat No. Z0458, Agilent Dako, Santa Clara, CA), 20S proteasome core subunits (1:1000, rabbit polyclonal, Cat No. PW8155, Enzo Life Sciences, Farmingdale, NY, USA) or CHOP (1:50, rabbit polyclonal, Cat No. sc-575, Santa Cruz, Santa Cruz, CA). For negative controls, either mouse IgG1 or rabbit immunoglobulin fraction (Agilent Dako, Santa Clara, CA, USA) was used as a substitution for a primary antibody. The control eyes published previously^19^ were used and the same images were displayed for this study (Fig. 5a).

### Real-time PCR

The retinas were isolated and immediately immersed in RNA later^®^ (Qiagen, Hilden, Germany). On the day of RNA extraction, each sample was transferred to a 2-ml tube containing a 0.5-ml Qiazol lysis reagent and a zirconia bead (Qiagen, Hilden, Germany), and rigorously homogenized for 5 min at 25 Hz using a TissueLyzer (Qiagen, Hilden, Germany). Total RNA was extracted individually from each retina, according to the rest of the protocol for an RNeasy 96 Kit provided by the manufacturer (Qiagen, Hilden, Germany). First strand cDNA was prepared using 0.2 μg (except for one experiment using 0.25 μg instead) of total RNA in a reagent mixture containing PrimeScript RT enzyme, oligo-dT primers and random 6 mers (PrimeScript RT reagent Kit, Takara, Shiga, Japan).

An aliquot of resultant cDNA was added to a master mix of either QuantiTect or QuantiFast Multiplex PCR kit (Qiagen, Hilden, Germany), and real-time PCR was performed using a 7500 Fast Real-Time PCR system (Applied Byosystems, Foster City) according to the manufacturer’s instructions. A pre-designed primer-probe mixture for Gapdh (Applied Byosystems, Foster City, CA, USA) or Nfl (Sigma-Aldrich, St. Louis, MO, USA) was used for this reaction. The sequences of forward and reverse primers used for Nfl were 5′-ACAAGCAGAATGCAGACATCA-3′ and 5′-GGAGGTCCTGGTACTCCTTC-3′, respectively, and the sequence of TaqMan^®^ probe was [FAM] 5′- CCATCTCGCTCTTCGTGCTTCGC -3′ [BHQ-1]. The sequences of primers and probe for Gapdh are not available, because they are kept undisclosed by the manufacturer. The real-time PCR conditions were the cycling conditions of 50 °C for 2 min and 95 °C for 15 min followed by 40 cycles of 94 °C for 1 min and 60 °C for 1 min. Fluorescence intensity at every annealing step was captured and threshold cycle time (C_T_) values was determined using the 7500 software. The comparative C_T_ method was deployed to determine Nfl expression relative to that of Gapdh. The relative Nfl expression levels were further normalized to the respective control (uninjected or vehicle group).

For the other retinal marker genes (see Supplementary Fig. S2), real-time PCR was performed using QuantiFast SYBR Green PCR Kit (Qiagen, Hilden, Germany) according to the manufacturer’s instructions. To quantify the expression levels of each target gene, a standard curve was generated using serial dilutions of a master cDNA mixture, when real-time PCR was performed for unknown samples. The relative expression level for a target gene in each sample was determined using the standard curve, and further normalized to that for Gapdh in the same sample. The sequences of primers and probes for those genes are shown in Supplementary Table S1.

### Statistical analysis

Each value represents the mean ± S.D. Graphpad Prism software (Graphpad Software, San Diego, CA, USA) was used for statistical analysis. Two groups were compared by either two-tailed paired t-test or F-test followed by two-tailed unpaired t-test. For comparisons among 3 groups and more, Brown-Forsythe test was initially performed to see if the variances are equal among all the groups. If not significant, one-way analysis of variance (ANOVA) was performed. Post-hoc analysis was then performed using Dennett’s multiple comparison test between the control and each treatment group, and Tukey’s test among all groups. If the variances were significantly different, nonparametric tests, namely, Kruskal Wallis and Dunn’s tests, were performed. For time-course analysis, two-way ANOVA was performed and followed by Sidak’s multiple comparison test. Differences were assumed to be statistically significant when *P* < 0.05.

## Supplementary information

Supplementary information file.

## Data Availability

All data generated or analysed during this study are included in this published article (and its Supplementary Information files).
