# Supplementary material for: Rotenone-induced inner retinal degeneration via presynaptic activation of voltage-dependent sodium and L-type calcium channels in rats
Source: Sci Rep. 2020 Jan 22;10:969. doi: 10.1038/s41598-020-57638-y (PMC6976703; doi:10.1038/s41598-020-57638-y)
Supplement: Supplementary file 1 — Supplementary information file. [file 41598_2020_57638_MOESM1_ESM.pdf]

Rotenone-induced inner retinal degeneration via presynaptic activation of voltage-dependent sodium and L-type calcium channels in rats

Masaaki Sasaoka[1], Takashi Ota[1] and Masaaki Kageyama[1]*

[1]Global Alliances and External Research, Santen Pharmaceutical Co., Ltd., Ikoma-shi, Nara 630-0101 Japan. All correspondces and requests for materials should be addressed to the corresponding author* (M.K., e-mail: masaaki.kageyama@santen.com)

Supplementary Fig. S1. Increased ubiquitin-, 20S proteasome- and CHOP-positive immunohistochemical staining in the rat retina exposed to rotenone. Rotenone (2 nmol/eye) was intravitreally injected, and eyes were enucleated 6 hrs following injection. Each cross-sectioned retina was subjected to immunohistochemical staining for antibodies against ubiquitin (a), 20S proteasome subunit (b) and CHOP (c). Each photograph shows the representative images of cross-sectioned retina following injection. Each scale bar shows 20 μm. Note that this experiment was performed as part of our previous study published elsewhere (Kageyama et al., PLoS One, 2019)[1]. See text and Fig. 5 for more details.

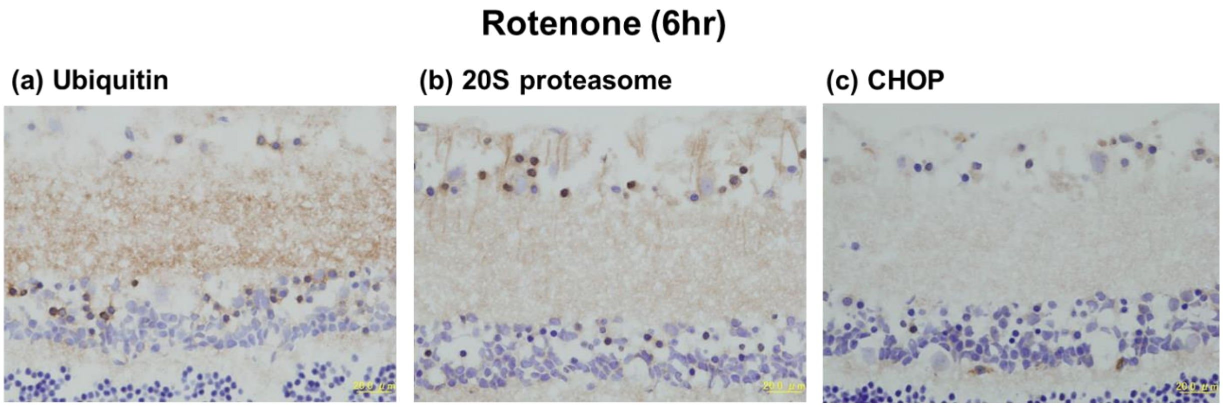

Supplementary Fig. S2. Rotenone-induced downregulation of retinal specific marker genes in the rat retina. Rotenone was intravitreally injected at doses of 0.4, 2 and 10 nmol/eye, and each eye was enucleated 24 hrs following injection. In the control group, the eyes were left uninjected. The retina was isolated from each eye and expression of each gene was determined by real-time PCR. The gene expression level was normalized to that of Gapdh in an individual retinal sample and is shown as the percentage of the uninjected control. (a) Amacrine cell markers: parvalbumin (Pvalb, open column, n=3 from 3 animals for untreated control; n=4 from 2 animals for rotenone) and tyrosine hydroxylase (Th, closed column, n=3 from 3 animals for untreated control; n=4 from 2 animals for rotenone); (b) bipolar cell markers: metabotropic glutamate receptor 6 (Grm6, open column, n=4 from 3 animals for untreated control; n=4 from 2 animals for rotenone) and protein kinase C alpha (Prkca, closed column, n=4 from 3 animals for untreated control; n=4 from 2 animals for rotenone); (c) photoreceptor marker genes: rhodopsin (Rho, open column, n=3 from 3 animals for untreated control; n=4 from 2 animals for rotenone) and opsin 1, short-wave-sensitive (Opn1sw, closed column, n=3 from 3 animals for untreated control; n=4 from 2 animals for rotenone). Each value represents the mean ± S.D. of 3-4 eyes from 2-3 animals. *P<0.05; **P<0.01; ***P<0.001, compared with the uninjected control by Dennett's or Dunn's multiple comparison test.

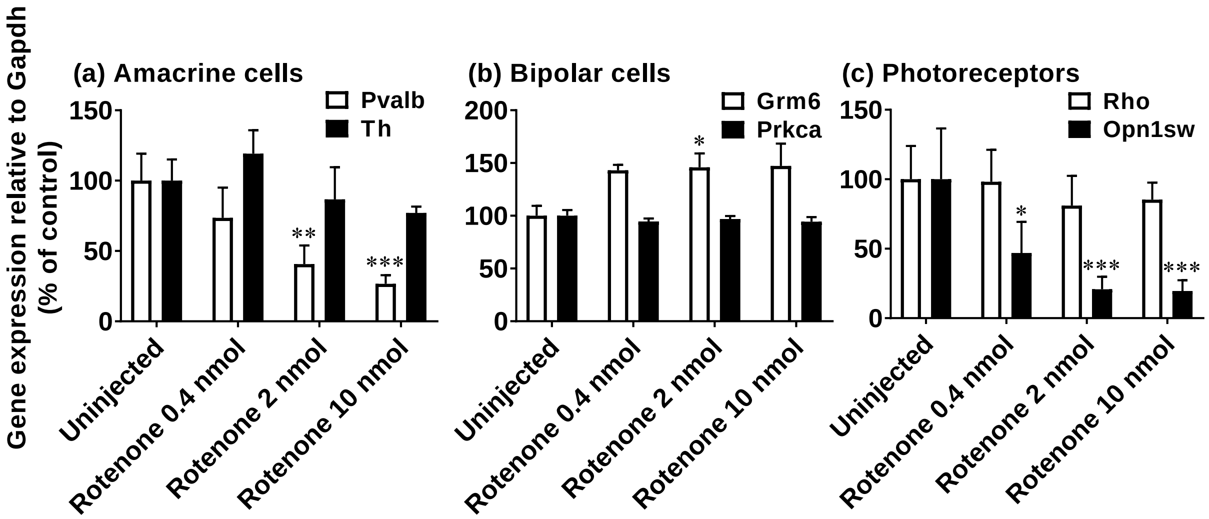

Supplementary Fig. S3. A diagram depicting the putative mechanisms underlying rotenone-induced inner retinal degeneration. Following intravitreal injection of rotenone, inhibition of complex I in the mitochondria (M) leads to increased reactive oxygen species (ROS). ROS inactivates Na^+-K^+ ATPase, resulting in presynaptic membrane depolarization[2]. Membrane depolarization activates voltage-dependent sodium channels and subsequently L-type calcium channels, allowing ions to enter the cytosol through these channels. ROS may directly activate both channels or through interacting with other protein kinases such as calcium/calmodulin kinase II. An increased intracellular calcium concentration stimulates glutamate (G) release via the Ca^{2+}-calmodulin (CAM) complex. Glutamate binds to and stimulates NMDA receptors, leading to activation of downstream events associated with cell death. The target sites of tested pharmacological agents are also shown. For more details, see the text.

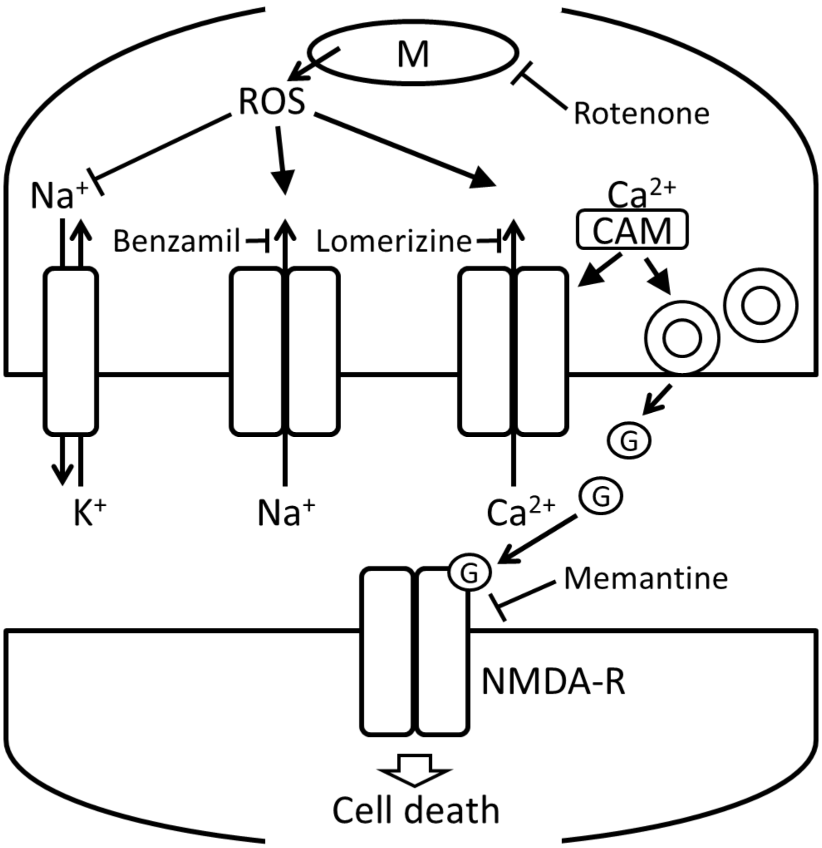

Supplementary Fig. S4. No protective effects of endoplasmic reticulum stress and unfolded protein response modulators against rotenone-induced inner retinal degeneration as measured by neurofilament light chain gene (Nfl) expression. Each modulator was premixed with 2 nmol/eye rotenone, and simultaneously injected into the vitreous of the rat eyes. In the control animals, the eyes were left uninjected. Twenty-four hours following injection, the retina was isolated and Nfl expression was determined by real-time PCR. The Nfl expression level was normalized to that of Gapdh in an individual retinal sample and is shown as the percentage of the respective control. (a) Uninjected (open column), rotenone alone (closed column), rotenone plus 12.5 nmol/eye salubrinal (dark grey), or 1 nmol/eye ansatrienin A (light grey). N=4 from 2 animals. (b) Uninjected (open column, n=4 from 2 animals), rotenone alone (closed column, n=5 from 3 animals), rotenone plus 1 nmol/eye TDZD-8 (dark grey, n=4 from 2 animals) or 20 nmol/eye TDZD-8 (light grey, n=4 from 2 animals). Each value represents the mean ± S.D. of 4 to 5 eyes from 2-3 animals. ***P<0.001, by Tukey's multiple comparison test.

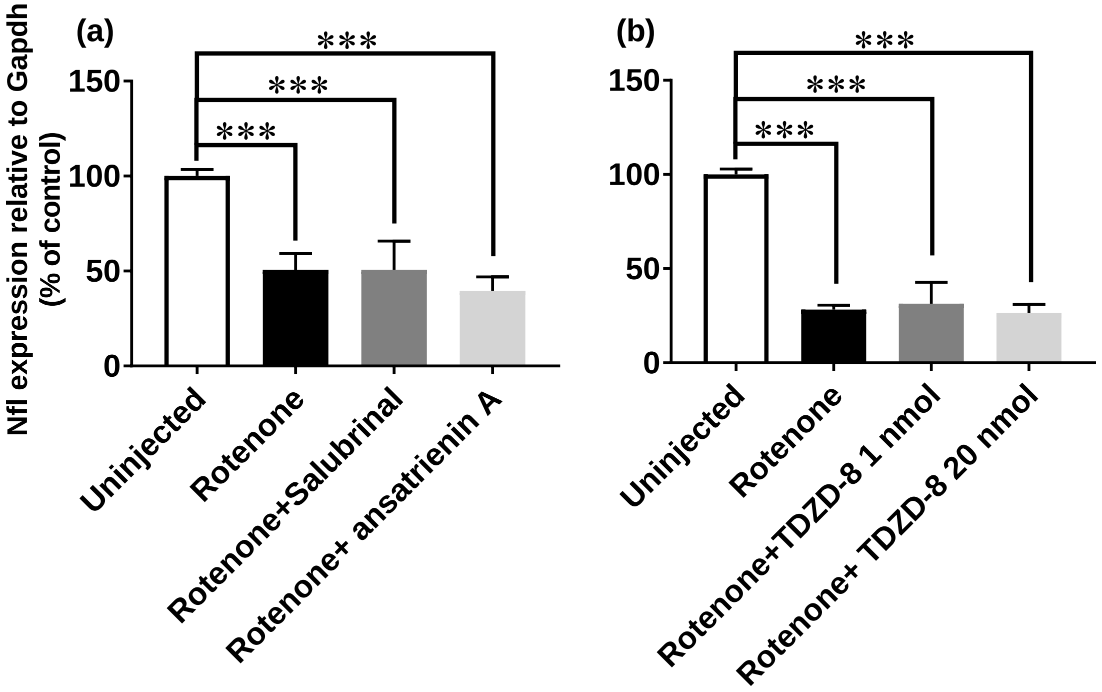

Supplementary Table S1. Sequences of primers for real-time PCR.

Gene symbol	Gene name (GenBank No.)	Primer sequence (5' to 3' orientation)	Size (bp)
Nfl	neurofilament, light polypeptide (NM_031783)	Forward: TACAGTGGCTTGCAGAGCAG Reverse: TCGTAGCCTCAATGGTCTCC	115
Pvalb	parvalbumin (NM_022499)	Forward: TGTTCCACATTCTGGACAAAGACAA Reverse: AGCAGACAAGTCTCTGGCATCTGA	101
Th	tyrosine hydroxylase (NM_012740)	Forward: CAGCTGTGCAGCCCTACCAA Reverse: TGTGTACGGGTCAAACTTCACAGAG	140
Grm6	glutamate receptor, metabotropic 6 (NM_022920)	Forward: TCTTTGGTACCGCCCAGTCAG Reverse: TCTGGATGGAACAGGATGACGTAG	136
Prkca	protein kinase C, alpha (NM_001105713)	Forward: undisclosed Reverse: undisclosed	-
Rho	rhodopsin (NM_033441)	Forward: ACACCTCACTGCATGGCTACTTTG Reverse: TTGCTCATGGGCTTGCAGAC	148
Opn1sw	opsin 1, short-wave-sensitive (NM_031015)	Forward: GCTGTACTTACGGCTTGTCACCA Reverse: CACACCATCTCCAGGATGCAG	120
Gapdh	glyceraldehyde-3-phosphate dehydrogenase (NM_017008)	Forward: GACAACTTTGGCATCGTGGA Reverse: ATGCAGGGATGATGTTCTGG	112

These primers were designed and provided by either Qiagen or Takara.

References

1 Kageyama, M., Ota, T., Sasaoka, M., Katsuta, O. & Shinomiya, K. Chemical proteasome inhibition as a novel animal model of inner retinal degeneration in rats. *PLoS One* **14**, e0217945, doi:10.1371/journal.pone.0217945 (2019).

2 Wagner, S., Rokita, A. G., Anderson, M. E. & Maier, L. S. Redox regulation of sodium and calcium handling. *Antioxid Redox Signal* **18**, 1063-1077, doi:10.1089/ars.2012.4818 (2013).
